# Cost-analysis of XELOX and FOLFOX4 for treatment of colorectal cancer to assist decision-making on reimbursement

**DOI:** 10.1186/1471-2407-11-288

**Published:** 2011-07-09

**Authors:** Vicki C Tse, Wai Tong Ng, Victor Lee, Anne WM Lee, Daniel TT Chua, June Chau, Sarah M McGhee

**Affiliations:** 1 Department of Community Medicine, School of Public Health, The University of Hong Kong, Hong Kong; 2 Department of Clinical Oncology, Pamela Youde Nethersole Eastern Hospital, Hong Kong; 3 Department of Clinical Oncology, The University of Hong Kong, Queen Mary Hospital, Hong Kong

## Abstract

**Background:**

XELOX (capecitabine + oxaliplatin) and FOLFOX 4 (5-FU + folinic acid + oxaliplatin) have shown similar improvements in survival in patients with metastatic colorectal cancer (MCRC). A US cost-minimization study found that the two regimens had similar costs from a healthcare provider perspective but XELOX had lower costs than FOLFOX4 from a societal perspective, while a Japanese cost-effectiveness study found XELOX had superior cost-effectiveness. This study compared the costs of XELOX and FOLFOX4 in patients with MCRC recently treated in two oncology departments in Hong Kong.

**Methods:**

Cost data were collected from the medical records of 60 consecutive patients (30 received XELOX and 30 FOLFOX4) from two hospitals. Drug costs, outpatient visits, hospital days and investigations were recorded and expressed as cost per patient from the healthcare provider perspective. Estimated travel and time costs were included in a societal perspective analysis. All costs were classed as either scheduled (associated with planned chemotherapy and follow-up) or unscheduled (unplanned visits or admissions and associated tests and medicines). Costs were based on government and hospital sources and expressed in US dollars (US$).

**Results:**

XELOX patients received an average of 7.3 chemotherapy cycles (of the 8 planned cycles) and FOLFOX4 patients received 9.2 cycles (of the 12 planned cycles). The scheduled cost per patient per cycle was $2,046 for XELOX and $2,152 for FOLFOX4, while the unscheduled cost was $240 and $421, respectively. Total treatment cost per patient was $16,609 for XELOX and $23,672 for FOLFOX4; the total cost for FOLFOX4 was 37% greater than that of XELOX. The addition of the societal costs increased the total treatment cost per patient to $17,836 for XELOX and $27,455 for FOLFOX4. Sensitivity analyses showed XELOX was still less costly than FOLFOX4 when using full drug regimen costs, incorporating data from a US model with costs and adverse event data from their clinical trial and with the removal of oxaliplatin from both treatment arms. Capecitabine would have to cost around four times its present price in Hong Kong for the total resource cost of treatment with XELOX to equal that of FOLFOX4.

**Conclusion:**

XELOX costs less than FOLFOX4 for this patient group with MCRC from both the healthcare provider and societal perspectives.

## Background

Increasing healthcare expenditure is a universal problem facing the developed world. In 2008, Australia's healthcare expenditure reached AUS$104 billion (8.5% of GDP) [1] and that of the United States (US) US$2.3 trillion (16% of GDP) [2]. Expenditure on drugs has recently been the fastest growing component of expenditure in Australia [1], Canada [3,4], UK [5] and US [6,7]. Whilst new treatments usually have a higher purchase price than older, possibly off-patent drugs, they may be a more efficient treatment when the full costs and consequences are taken into account. This is particularly the case with new, targeted therapies in oncology, which often have high drug-acquisition costs [8-10]. Cost-effectiveness analyses (CEA) are therefore increasingly used by decision-makers to determine which drugs should be included in public formularies [1,3-7,11-17]. The UK National Institute of Health and Clinical Excellence has used CEA since 1999 [14,18], Australian Pharmaceutical Benefits Advisory Committee since 1993 [14,19] and Canadian Common Drug Review since 2002 [14,20]. In Hong Kong, the Hospital Authority (HA) is a government-funded provider of specialist healthcare services. Its Drug Advisory Committee appraises new drugs for inclusion into the HA Drug Formulary but has not, to date, required cost-effectiveness evidence as a component of drug evaluation [21,22]. This is a common situation for smaller countries or those, for example, in the Asia Pacific region where skills in health economics are lacking [22,23] and for which the available cost-effectiveness information, derived from other countries, may or may not be applicable [22,24,25].

In this paper we demonstrate how the lack of cost-effectiveness information could mask the cost implications of treatment decisions. The two treatments compared were available for use in Hong Kong in the treatment of metastatic colorectal cancer (MCRC) but one (capcetabine + oxaliplatin) was totally self-financed by the patient while the other had two components (5-FU + folinic acid) subsidised by the HA although the other component (oxaliplatin) was self-financed. By examining local data on costs, we were able to demonstrate that the current reimbursement procedures are probably not the most efficient choice for the HA.

## Methods

### The treatments compared

In improving survival of patients with MCRC, XELOX (capecitabine + oxaliplatin) has been shown in several trials to be as effective as FOLFOX4 (5-FU + folinic acid + oxaliplatin) [26-28]. Economic models taking a provider's perspective have showed XELOX to be more cost-effective than FOLFOX4 in Japan [29] but of equivalent cost-effectiveness in US [30]. However, the US model demonstrated cost-effectiveness of XELOX when a societal perspective, including patient costs and lost productivity, was taken.

### The economic model

Cost-effectiveness analysis estimates a cost per unit of benefit for alternative treatments so a treatment which provides more benefit at the same cost or the same benefit at lower cost would be more efficient. However, if two treatments provide identical benefits, then the one which costs least is more efficient. Since the clinical trials had shown that XELOX was at least as effective as FOLFOX4, [26-28] we focused on the costs of the two treatments to determine the relative efficiency in Hong Kong, assuming that the clinical effects would be the same as in the US studies. While it is usual to accept data on effectiveness as being applicable to a new population, the same cannot be done with costs and resource use which are likely to vary because of local prices, values, treatment protocols and health care practices.

### The patients sampled

Patients with previously untreated MCRC who received either XELOX or FOLFOX4 in two large general hospitals (Queen Mary Hospital and Pamela Youde Nethersole Eastern Hospital) in Hong Kong were retrospectively identified from the records of the oncology departments. The predefined earliest treatment start date was January 2003 to ensure comparability of other factors and we aimed for thirty patients to be identified retrospectively as undergoing each treatment. In practice the earliest start date was January 2004 and the latest was December 2008. Information on all use of public hospital inpatient or outpatient resources was collected from the medical records by experienced researchers for up to 28 days after the end of chemotherapy treatment. This included hospital days, all outpatient visits and associated investigations and treatments. Patients' baseline demographics and clinical characteristics were compared across both groups using a t-test for means and the Kruskal-Wallis rank test for frequencies.

### Identification, measurement and valuation of cost data

Monetary values of health care resources used were taken from the Hong Kong Government Gazette, the official source for charges to non-entitled persons for use of public hospital services i.e. a cost-recovery charge. The Gazette dates from 2003 but had not been updated since then and so applied in 2009. Hong Kong citizens are charged only a small co-payment for public inpatient and outpatient services with the exception of drugs which fall outside of the drug formulary which can be prescribed on a self financed basis. The costs of capecitabine and oxaliplatin for those on the XELOX regimen and oxaliplatin for those on the FOLFOX4 regimen were borne by patients because these drugs are not in the formulary [31]. All costs have been reported in US$ (1US$ = 7.78HK$) to the nearest $ and 2009 is used as the base year for costing.

In order to identify differences in resource use for treatment and that for possible adverse events, all resources used were separated into scheduled (defined as incurred for chemotherapy delivery or standard follow-up) and non-scheduled. Non-scheduled costs would include any other costs such as treatment for adverse events, disease progression or costs unrelated to the chemotherapy.

From the information in the medical records, a hospital stay for chemotherapy delivery of 1 day for XELOX and 3 days for FOLFOX4 was normal. Any further days in hospital were therefore classed as unscheduled costs. Each hospital day was valued using the average bed-day cost of $424 [32]. However, in order not to overestimate the cost of the hospital days used for chemotherapy, the single hospital day used for XELOX was valued at $180, which is the listed cost of a one-day admission in a community hospital plus an intravenous administration of drugs [32]. For FOLFOX4, the 3 days for chemotherapy were valued as the cost for two days or overnight stays i.e. $848. This gave a conservative estimate for FOLFOX4 and a probably realistic estimate for XELOX.

Outpatient visits were valued using the average cost of a specialist visit ($77), a GP visit ($26) or a visit to an Accident and Emergency Department ($73) [32]. All investigations and treatments were valued using the standard costs for each [33]. All drugs were costed using the standard drug costs in 2009 [34] and categorized as chemotherapy regimens or non-chemotherapy drugs.

### Societal costs

Travel and time costs were estimated as this information was not recorded in the medical records. Outpatient visits were assumed to take 2 hours per visit including travel and were valued at $5 per hour based on the median monthly salary [35]. Travel costs were based on the actual distance between the patient's home address and the hospital and the taxi fare for that distance [36] giving an average round-trip value of $14 for the XELOX group and $12 for the FOLFOX4 group. Time costs were also applied to the time spent in hospital assuming that the day admission for XELOX took 6 hours and for FOLFOX4 took 2 days (48 hours). For unscheduled hospital visits, the average number of days was multiplied by 24 hours to generate the average number of hospital hours and travel costs were valued as for outpatient visits.

To estimate a confidence interval for the total costs of the two drug regimens, we carried out a bootstrap procedure with 1,000 replications, sampling with replacement from the original data.

### Sensitivity analysis

Sensitivity analyses were performed to evaluate the impact of (a) using the costs of a full dose of each chemotherapy drug based on the average body surface area of 1.65 m^2 ^rather than the actual, lower dose recorded in the records and the full number of cycles as per the protocol i.e. 8 cycles for XELOX and 12 for FOLFOX4; (b) with the cost of oxaliplatin removed because it is an expensive, self-financed part of both chemotherapy regimens and (c) using a US model developed in Excel by Garrison et al [30] into which the Hong Kong unit costs were inserted using an exchange rate of HK$7.78 to US$1. This US model compared the scheduled costs of XELOX versus FOLFOX4 arms in a non-inferiority study which found XELOX to be at least as effective as FOLFOX4. The study was conducted in a US setting from both payer and societal perspectives and included resource use, frequency and treatment of adverse events. Although all unit costs used were based on Hong Kong data, the frequency of adverse events and loss of working time were based on the US study.

Finally, in order to determine the sensitivity of the results to the cost of capecitabine, we varied the average chemotherapy cost for the XELOX group until the total costs were the same for the XELOX and FOLFOX4 groups.

Ethics approval for the study was obtained from the Institutional Review Board of both hospitals.

## Results

### Patients' baseline characteristics

The baseline characteristics of patients receiving XELOX and FOLFOX4 are shown in Table 1. There were no statistically significant differences in the recorded characteristics except that those in the XELOX group were more likely to have had liver metastases at baseline.

**Table 1:** Patients' baseline characteristics: XELOX and FOLFOX4 groups

		XELOX (n = 30)	FOLFOX4 (n = 30)	Statistical significance p-value
Male, %		57	57	
Chinese ethnicity, %		94	100	
Mean age, years		63.9	61.9	0.936*
Mean weight, kg		61.6	59.8	0.544*
Mean height, cm		165.4	162.6	0.281*
Mean body surface area, m^2^		1.65	1.62	0.555*
ECOG PS, % of patients	0	34	30	0.860^‡^
	1	53	53	
	2	4	4	
Alkaline phosphatase, % abnormal		20	17	0.741^‡^
No. of metastatic sites, % of patients	1	57	53	0.822^‡^
	2	33	37	
	≥3	10	10	
Liver metastases present, % of patients		77	50	0.034^‡^
Status at August 2009, % surviving		67	47	0.121^‡^

* T-test, ^‡ ^Kruskal-Wallis rank test.

### Planned and actual dose

The average number of cycles of chemotherapy delivered was 7.3 out of a planned 8 cycles for XELOX and 9.2 out of a planned 12 cycles for FOLFOX4. Overall, 91% of the planned amount of capecitabine and 89% of oxaliplatin was administered to the XELOX patients while 77% of the planned amount of 5-FU, folinic acid and oxaliplatin was administered to the FOLFOX4 patients.

### Healthcare use

The average follow-up period, including the chemotherapy treatment and up to 28 days post-treatment, was 199 and 168 days for the XELOX and FOLFOX4 groups respectively. The healthcare use in the XELOX and FOLFOX4 chemotherapy groups within the follow-up period is shown in Table 2. The average number of hospital days for chemotherapy in the FOLFOX4 group was more than three times that of the XELOX group (33.2 vs. 10.3 days). The total cost per patient was higher for the FOLFOX4 group compared with the XELOX group both for the scheduled costs (chemotherapy plus expected hospital days for its administration and scheduled clinic visits) and for the unscheduled costs (Table 3). Hence, in this analysis FOLFOX4 was 43% more expensive than XELOX. The bootstrapped mean total costs and 95% confidence interval were $16,410(95%CI: $14,976-18,019) for XELOX and $22,169 (95%CI: $20,010-24,315) for FOLFOX4.

**Table 2:** Healthcare use in MCRC patients receiving XELOX or FOLFOX4

	XELOX (n = 30) Mean no./person (SD)	FOLFOX4 (n = 30) Mean no./person (SD)
Chemotherapy cycles	7.3 (1.4)	9.2 (2.8)

Other drugs	9.3 (5.8)	12.4 (7.2)
Hospital bed-days	10.3 (5.2)	33.2 (9.1)
Outpatient clinic visits	15.5 (3.4)	16.5 (5.1)
Laboratory investigations	38.6 (11.2)	37.3 (12.8)
X-rays and scans	4.3 (3.1)	3.8 (2.5)

**Table 3:** Comparative costs (US$) for first-line treatment of MCRC with XELOX and FOLFOX4 from a healthcare payer perspective

		XELOX (n = 30) Mean (SD)	FOLFOX4 (n = 30) Mean (SD)
Scheduled costs per cycle			
Chemotherapy cost		1,184 (152)	724 (84)
Hospital days for chemotherapy delivery		180 (-)*	848 (-)*
Hospital tests		55 (106)	76 (163)
Outpatient visits		155 (52)	135 (46)
Outpatient tests at scheduled visits		472 (291)	368 (191)

Total scheduled cost per cycle		2,046 (325)	2,152 (307)
Total scheduled cost per patient		14,866 (3,823)	19,801 (5,405)

Other unscheduled costs			
Drug costs		4 (7)	44 (191)
Hospital days		201 (355)	355 (670)
Outpatient visits		15 (21)	13 (39)
Outpatient tests at unscheduled visits		20 (53)	9 (28)

Total unscheduled cost per cycle		240 (364)	421 (890)

Total unscheduled cost per patient		1,743 (2,197)	3,871 (3,870)

Total cost per patient		16,609 (4,710)	23,672 (5,904)

* These are estimated rather than measured (see methods) therefore no SD is calculated.

It was assumed that treatment costs for both related and unrelated adverse events (AE) would be captured as unscheduled costs. The most common AE observed in both groups was nausea; the most common AE in the FOLFOX4 group was neutropenia followed by thrombocytopenia and mild diarrhoea whilst the most common AE in the XELOX group was mild hand foot syndrome followed by mild peripheral neuropathy and thrombocytopenia.

### Societal perspective

The scheduled patient time cost for XELOX was $51 per cycle with the unscheduled time cost $59. Scheduled and unscheduled travel costs were $42 and $17 per cycle respectively giving a total societal cost per patient for XELOX of $17,836 (Table 4). For FOLFOX4, the scheduled patient time costs were $265 per cycle and the unscheduled costs $99 while scheduled and unscheduled travel costs were $33 and $14 per cycle respectively. This gave a total treatment cost per patient for FOLFOX4 from a societal perspective of $27,455 (Table 4). Thus, the inclusion of societal costs further increased the cost difference between the two drugs with FOLFOX4 now being 54% more expensive than XELOX.

**Table 4:** Comparative costs (US$) for first-line treatment of MCRC with XELOX and FOLFOX4 from a societal perspective

		XELOX (n = 30) Mean (SD)	FOLFOX4 (n = 30) Mean (SD)
Scheduled costs per cycle			
Treatment cost		2,046 (325)	2,152 (307)
Patient time cost		51 (-)*	265(-)*
Patient travel cost		42 (-)*	33 (-)*

Total scheduled cost per cycle		2,139	2,450
Total scheduled cost per patient		15,544	22,539

Other unscheduled costs			
Treatment cost		240 (364)	421 (890)
Patient time cost		59 (-)*	99 (-)*
Patient travel cost		17 (-)*	14 (-)*

Total unscheduled cost per cycle		315	534

Total unscheduled cost per patient		2,292	4,915

Total cost per patient		17,836	27,455

* These are estimated rather than measured (see methods) therefore no SD is calculated.

### Sensitivity analysis

Full dosage based on the average body surface area would cost $1,367 for XELOX and $751 for FOLFOX4 and, assuming the complete 8 or 12 cycles respectively had been administered, total costs per patient would become $19,753 for XELOX and $31,200 for FOLFOX4 i.e. FOLFOX4 was 58% more expensive than XELOX.

Removal of oxaliplatin from the analysis did not impact on the cost difference from a healthcare payer perspective since FOLFOX4 was now 69% more expensive than XELOX.

XELOX was cost-effective in the US from a societal perspective but not from a payer perspective [30]; however, using the same model with cost data from Hong Kong showed XELOX to be cost-effective from both payer and societal perspectives.

For the total average cost in the XELOX group to equal that in the FOLFOX4 group, the average cost of the capecitabine component of the chemotherapy would have to be around $1,090 i.e. over four times the $213 found in the study.

## Discussion

The XELOX regimen comprising capecitabine and oxaliplatin requires less intravenous administration than FOLFOX4, comprising 5-FU, folinic acid and oxaliplatin. Therefore it might be expected that it would cost less overall when costs of drug delivery are taken into account. This study showed that this was indeed the case and that, when the fewer cycles of therapy required with XELOX (8 versus 12) were also taken into account, the savings in other costs outweighed the higher purchase price of the XELOX regimen. However, the publicly funded provider in HK subsidised the use of FOLFOX4 and not XELOX, thus encouraging the use of a regimen that resulted in higher use of hospital resources. Those patients willing to bear the cost of the alternative therapy because of the benefits they perceived to themselves were saving resource costs for the provider. This is, of course, because historically FOLFOX4 has been the approved treatment and XELOX, the newer treatment, had a higher purchase price per dose and had not yet been considered for inclusion into the list of approved drugs for subsidy.

This cost analysis was a relatively simple study designed to obtain information to allow a more considered assessment of the two regimens. Therefore, it must be asked whether the information collected is adequate for decision-making. Cost minimisation analysis (CMA) is defined by Drummond et al [37] as a design used where the consequences are the same with a disparity in costs only and it is relatively low cost, simple and quick to conduct. This is an advantage for countries which have few people with economic modelling skills. However, it depends on the major assumption that the clinical effects of the comparison treatments are the same. Such a finding of similar effectiveness is often extrapolated from 'negative' trials, i.e. where neither drug is found to be superior but these studies may have insufficient power to test equivalence of effects [38]. In the case of these two drugs, there was some evidence from trials designed to determine that the new drug was not inferior to the alternatives [26-28] but we did not subject that evidence to a full review. One concern, when the benefits have not been included in the economic modelling, is that any negative impacts of the drugs may not have been fully accounted for [35]. Therefore in a costing study such as this one, it would be important to consider what negative outcomes might occur in the particular clinical situation and to consider doing a full cost-effectiveness study if there should be any doubts. For example, with a treatment such as XELOX which is taken on an outpatient basis, we might be concerned that reduced frequency of contact with the doctor could delay identification of side effects. Careful monitoring of the patients or specific patient advice plus a contact number to call might help to resolve this question.

A cost analysis is highly dependent on good local data on costs and sufficiently completed medical records from which resource use can be extracted. Hong Kong has a computerised clinical information system which covers all public medical services; cross-referencing was conducted in this study between different sections of the record to ensure completeness of the data captured.

We did not apply any discounting in this study because the time course for each patient was less than a year. All costs relate to the base year of 2009 even though the actual resource use was in the years between 2004 and 2008.

This study found that, despite XELOX having a 64% higher purchase price than FOLFOX4, from the provider's perspective it could be a more cost-effective option in Hong Kong since the total cost of its use was 70% of that of FOLFOX4 when all healthcare resource use was taken into account. This was mainly because of the reduced need for hospital-based intravenous drug delivery, a finding that is consistent with Garrison et al [30] and Scheithauer et al [39] in comparisons of the same two drug regimens, as well as Perrocheau et al [40] when comparing XELOX against FOLFOX6.

The finding of lower cost for XELOX differs from the US results where the XELOX regimen was more expensive than FOLFOX4. This is mainly the result of capecitabine being approximately 8 times the cost of 5-FU in US while in Hong Kong it was just over twice the price. Our sensitivity analysis also showed that capecitabine would have to be four times as expensive for the total resource cost in the XELOX group to equal that in the FOLFOX group.

The relative differences in other costs also combine to make XELOX less costly even when the US data on side-effects and subsequent resource use were modelled together with Hong Kong costs. Taking a societal perspective increased the cost difference between XELOX and FOLFOX4 as also found in the US [30]. This study demonstrates the fact that cost-effectiveness evidence cannot be easily generalised across jurisdictions [22-25,41-43] and that local cost data are required.

We estimated the total costs of the treatment regimens and this should now provide very useful information for the healthcare providers to determine the impact on their budgets of using one drug rather than the other. Of course, the total costs of a treatment may be spread across different budgets, for example a drug procurement budget and a hospital bed provision budget, including staffing, testing and other items. While there may be higher costs in one budget e.g. drug procurement this might be more than compensated for by decreases in another. We hope that the information provided would allow such specific budget impacts to be estimated. We also hope that our study will encourage decision makers to take a more comprehensive view of costs which might encompass more than one budget, rather than focussing solely on the price of the drug.

### Limitations of This Paper

Our study was a retrospective and non-randomised study. It reflects the actual resource use of the subjects' care but, of course, is subject to bias. Allocation to the specific treatment might have been biased by some patient characteristics and, since one drug was substantially self-paid and the other one subsidised, the socio-economic status of the patients could have differed. However we had no information on subjects' socio-economic status for comparison. On the other hand, the comparison of subjects' characteristics found a statistically significant difference only in the proportion with liver metastases and the number was higher in the XELOX group, perhaps increasing the costs in that group. However, with only 30 subjects in each group, the statistical power of such comparisons is low and so we cannot rule out the possibility of some bias in treatment allocation.

Due to the greater number of patients treated on XELOX than FOLFOX4 in Hong Kong in recent years it was not possible to match the two chemotherapy groups in terms of treatment years so some FOLFOX4 patients' treatments date back to 2004-2005 while all XELOX patients were 2006-2008. However, we do not believe that the clinical management practices have changed much over this time period.

We may have underestimated the benefits of XELOX. Shiroiwa et al [29] compared the two drug regimens in Japan using utility values collected during the clinical trials and estimated quality-adjusted progression-free survival days. They demonstrated better quality of life in the XELOX group which they said enhanced the regimen's cost-effectiveness relative to FOLFOX4.

We had no information on costs of carers and have therefore underestimated the societal costs but including these would predictably make XELOX even less costly than FOLFOX4 because of the time spent attending hospital by the carer as well as the patient.

## Conclusion

We found that using XELOX cost less than using FOLFOX4 when all hospital resource use was considered. The principal reason for this lower cost was the reduced use of hospital bed-days for the treatment administration. Taken along with the results of clinical trials, this would imply that XELOX is a cost-effective option in Hong Kong. Despite the weaknesses of costing studies, they can have a role in showing that the impact on other resources, in this case hospital bed-days and costs of IV administration, can sometimes more than compensate for higher drug acquisition costs. This may be particularly important in oncology where tremendous advances are being made but the purchase price of the new drugs is often very high.

## Competing interests

Dr. Vicki C Tse, June Chau and Prof. Sarah M. McGhee report receiving research funding from Roche Hong Kong. Drs. Wai Tong Ng, Victor Lee, Anne WM Lee and Daniel TT Chua have no competing interests to declare.

## Authors' contributions

WTN, VL, AWML, DTTC, and SMMG contributed to the study conception and design. All authors were involved in data acquisition and/or data analysis and interpretation. All authors contributed to drafting, editing or critical revision of the manuscript and all read and approved the final manuscript.

## Pre-publication history

The pre-publication history for this paper can be accessed here:

http://www.biomedcentral.com/1471-2407/11/288/prepub
